# High *NLRC5* Expression Is Associated with an Immunosuppressive Tumor Microenvironment and Poor Prognosis in Esophageal Squamous Cell Carcinoma

**DOI:** 10.3390/cancers18071117

**Published:** 2026-03-30

**Authors:** Heng Xiao, Jingyue Fan, Jinyun Zhang, Caixia Cheng, Bin Song, Ling Zhang, Yanghui Bi, Xiaolong Cheng

**Affiliations:** 1 Translational Medicine Research Center, Department of Pathology & Shanxi Key Laboratory of Carcinogenesis and Translational Research of Esophageal Cancer, Shanxi Medical University, Taiyuan 030001, China; 2 Key Laboratory of Cellular Physiology of the Ministry of Education, Department of Pathology, Shanxi Medical University, Taiyuan 030001, China; 3 Shanxi Bethune Hospital, Shanxi Academy of Medical Sciences, Third Hospital of Shanxi Medical University, Tongji Shanxi Hospital, Taiyuan 030032, China; 4 Shanxi Province Cancer Hospital/Shanxi Hospital Affiliated to Cancer Hospital, Chinese Academy of Medical Sciences/Cancer Hospital Affiliated to Shanxi Medical University, Taiyuan 030001, China; 5 Department of Pathology, The First Hospital, Shanxi Medical University, Taiyuan 030001, China

**Keywords:** ESCC, *NLRC5*, T-cell exhaustion, PANoptosis, tumor microenvironment

## Abstract

**Background:** Immunotherapy efficacy in esophageal squamous cell carcinoma (ESCC) is often limited by an immunosuppressive tumor microenvironment (TME). NLRC5, a key regulator of MHC-I antigen presentation, exhibits context-dependent roles in tumor immunity; however, its function in ESCC remains unclear. This study aimed to systematically investigate the expression pattern, prognostic value, and immunological role of NLRC5 in ESCC. **Methods:** An integrated analysis of bulk RNA sequencing and single-cell RNA sequencing (scRNA-seq) data was performed using multiple cohorts, including The Cancer Genome Atlas, Gene Expression Omnibus, and an in-house ESCC cohort. Differential expression, survival analysis, immune infiltration estimation, and functional enrichment analyses were conducted to elucidate the role of NLRC5 in the tumor microenvironment. **Results:** NLRC5 was significantly upregulated in ESCC and its high expression independently predicted poor patient survival. Although NLRC5 expression was associated with increased CD8^+^ T cell infiltration, it was paradoxically accompanied by features of T-cell exhaustion and elevated expression of multiple immune checkpoints. Moreover, NLRC5-high tumors were enriched in transcriptional programs related to PANoptosis, indicating an additional immunosuppressive mechanism within the TME. **Conclusions:** NLRC5 is not only a prognostic biomarker but also a key modulator of an immune-active yet functionally suppressed tumor microenvironment in ESCC. These findings highlight NLRC5 as a potential therapeutic target for restoring effective antitumor immunity.

## Simple Summary

Esophageal squamous cell carcinoma is an aggressive cancer with limited treatment success, and many patients do not respond well to immunotherapy because tumors can weaken immune function. In this study, we investigated the role of *NLRC5*, a gene involved in immune regulation, in shaping the tumor immune environment. By analyzing data from multiple patient cohorts and examining tumors at both tissue and single-cell levels, we found that *NLRC5* is frequently increased in this cancer and that higher levels are associated with poorer survival. Although tumors with elevated *NLRC5* contain more immune cells, these cells show features of functional exhaustion and increased expression of inhibitory molecules, suggesting that they are unable to effectively attack the tumor. Our findings indicate that *NLRC5* may serve as a useful marker for prognosis and a potential therapeutic target to improve immune-based treatments in this disease.

## 1. Introduction

Esophageal squamous cell carcinoma (ESCC) remains one of the most lethal malignancies worldwide, with a 5-year survival rate below 30% [1,2].The poor prognosis is attributed to late diagnosis, limited therapeutic options, and an incomplete understanding of its molecular underpinnings [3,4]. While immunotherapies, such as immune checkpoint inhibitors, have revolutionized cancer treatment, their efficacy in ESCC is often suboptimal [5,6]. A major barrier is the establishment of an immunosuppressive tumor microenvironment (TME) that fosters T-cell exhaustion and limits the activity of infiltrating immune cells [7,8,9]. Therefore, elucidating the mechanisms that drive this immunosuppressive landscape is critical for developing more effective therapeutic strategies.

NLRC5, a member of the NLR family containing a CARD domain, is widely recognized for its role in regulating MHC class I gene transcription [10,11,12,13,14]. However, emerging evidence points to its broader role in modulating inflammatory signaling and cell death pathways [15,16,17,18]. This functional versatility has led to complex and sometimes contradictory reports on its role in cancer. While its canonical function suggests it should enhance anti-tumor CD8^+^ T-cell immunity, recent studies have also linked *NLRC5* to the establishment of an immunosuppressive TME by promoting T-cell dysfunction [12,19]. The net effect of *NLRC5* on the immune landscape and clinical outcomes in ESCC, however, remains largely unexplored.

To further investigate this question, we carried out a comprehensive multi-omics study. We hypothesized that NLRC5 contributes to the regulation of the immune microenvironment in ESCC. By integrating bulk transcriptomic data from multiple independent cohorts, including TCGA and GEO (GSE53625), alongside our in-house ESCC cohort (CancerCell cohort, *n* = 155), we rigorously evaluate the clinical relevance of *NLRC5* in ESCC, dissect its relationship with the composition and functional state of the tumor immune infiltrate, and employed its potential involvement in broader immunosuppressive mechanisms such as immune cell death. Our findings establish *NLRC5* as a key negative regulator of anti-tumor immunity in ESCC, where its overexpression is associated with a TME rich in exhausted CD8^+^ T cells and signatures of PANoptosis, ultimately contributing to poor patient outcomes.

## 2. Materials and Methods

### 2.1. Clinical Samples

Three bulk ESCC cohorts were utilized in the present study. The HRA003107 transcriptome and methylation data from 155 ESCC samples and their paired normal tissues were obtained from our previous research [20]. Additionally, GSE53625 transcriptome data from 179 ESCC samples and their paired normal tissues were retrieved from the GEO database. Pan-cancer data and ESCC-specific data from TCGA were also included in the analysis. Methylation data from 12 samples (GSE52826) were also downloaded from GEO. Furthermore, single-cell RNA sequencing (scRNA-seq) data generated from our in-house cohort comprising different tissue types were included in the analysis.

### 2.2. Cell Culture

All cell lines, including immortalized esophageal epithelial cells and ESCC cell lines, were generously provided by the Shenzhen Bay Laboratory, Cancer Research Institute. The NE2 and NE3 cell lines were cultured in dKSFM (Gibco, thermo Fisher Scientific, Waltham, MA, USA, 10744-019), EpiLife (Gibco, M-EPI-500-CA), and EDGS (Gibco, S-012-5), and supplemented with 1% penicillin-streptomycin (Gibco, PS). ESCC cell lines were maintained in RPMI-1640 medium (Gibco, C11875500CP) supplemented with 10% FBS (ExCell, Shanghai, China, FSP500) and 1% PS (Gibco, PS) at 37 °C with 5% CO_2_. All cell lines were authenticated by short tandem repeat (STR) analysis, and no mycoplasma contamination was detected.

### 2.3. Functional Enrichment Analysis

The Pearson correlation coefficients between *NLRC5* and each gene within the respective cohorts were calculated. The most correlated genes or the feature gene lists of specific cell clusters were then uploaded to the DAVID database (v6.8) for annotation, visualization, and integrated discovery. We selected the official gene symbols as identifiers and specified Homo sapiens as the species. Subsequently, we obtained enrichment results for Gene Ontology (GO) analysis and the Kyoto Encyclopedia of Genes and Genomes (KEGG) pathway analysis. The top five results, ranked by ascending adjusted *p* values (Benjamini–Hochberg correction), were presented in this study. Pathways with FDR < 0.05 were considered statistically significant.

### 2.4. Methylation Analysis

Methylation and gene expression data, along with gene annotation files, were obtained from three cohorts: the CancerCell cohort, TCGA, and GSE52826. Corresponding gene annotation information was obtained using the biomaRt package in R. Unpaired tests were employed to compare the average methylation levels between normal and tumor tissues. The association between gene expression and DNA methylation levels was further examined using Spearman correlation analysis.

### 2.5. Immune Cell Infiltration Analysis

The ESTIMATE algorithm was applied to infer the proportions of stromal and immune components in tumor tissues based on gene expression signatures. This approach was used to evaluate the tumor microenvironment (TME) of ESCC samples across the three cohorts. Stromal score, immune score, ESTIMATE score, and tumor purity were calculated using the estimate R package [21].

CIBERSORT, a deconvolution algorithm, was employed to estimate the proportions of 22 immune cell types in each ESCC patient across the three cohorts based on expression profiles [22]. The sum of the 22 immune cell fractions for each sample was constrained to 1.

Additionally, using the single-sample gene set enrichment analysis (ssGSEA) method from the GSVA R package [23], we assessed the infiltration of 28 immune cell types according to the expression levels of genes from 28 published immune cell gene sets.

### 2.6. xCell Analysis

To further characterize the immune and stromal landscape of ESCC, we applied the xCell algorithm, a gene signature-based approach designed to infer the enrichment of multiple immune and stromal cell types. Using transcriptomic data from three cohorts, xCell was used to estimate the relative abundance of 64 cell populations in each sample. The results provided a detailed view of the tumor microenvironment (TME), including the proportions of various immune cell populations, stromal components, and their interactions. The analysis was conducted using the xCell R package, and we compared the cell type scores between tumor and normal tissues to identify significant differences in cell infiltration patterns across the cohorts.

### 2.7. TIDE Analysis

To assess T cell dysfunction and exclusion within the tumor microenvironment, we applied the Tumor Immune Dysfunction and Exclusion (TIDE) framework. TIDE integrates gene expression signatures to predict immune evasion mechanisms, particularly focusing on T cell dysfunction in tumors infiltrated with cytotoxic T lymphocytes (CTLs) and T cell exclusion in non-infiltrated tumors. By utilizing transcriptomic data from three ESCC cohorts, TIDE was employed to evaluate the extent of T cell dysfunction and exclusion for each patient.

### 2.8. Gene Set Enrichment Analysis (GSEA)

To explore the pathways associated with T cell exhaustion and programmed cell death mechanisms (apoptosis, necroptosis, and pyroptosis), we performed Gene Set Enrichment Analysis (GSEA) using three cohorts, including TCGA, CancerCell, and GSE53625 datasets. For the analysis, T cell exhaustion gene lists were obtained from the literature, and gene sets for apoptosis, necroptosis, and pyroptosis were obtained from the Molecular Signatures Database (MSigDB) (https://www.gsea-msigdb.org/gsea/msigdb, accessed on 20 October 2023). RNA-seq expression profiles of the TCGA, CancerCell, and GSE53625 cohorts were divided into *NLRC5*-high and NLRC5-low groups according to the median *NLRC5* expression level. GSEA was performed using the GSEA software (4.3.3).

### 2.9. Single-Cell RNA-Seq Data Processing and Correlation Analysis

Single-cell RNA sequencing (scRNA-seq) data were analyzed following a previously established analytical workflow. Raw sequencing reads were processed using the Cell Ranger pipeline (10x Genomics) to generate gene–cell expression matrices based on the GRCh38 reference genome.

Quality control filtering was performed to remove low-quality cells and genes prior to downstream analyses using commonly applied criteria. Batch effects across samples were corrected using the Harmony algorithm. Dimensionality reduction, clustering, and cell population identification were conducted using the Seurat package (v4).

To mitigate dropout events inherent to single-cell RNA-seq data, gene expression matrices were further processed using scImpute, which estimates missing expression values based on cell similarity. Imputation was performed using the default parameters recommended by the developers.

Following imputation, Pearson correlation analysis was conducted to evaluate the association between NLRC5 expression and immune checkpoint-related genes, including *PDCD1*, *ENTPD1*, *LAG3*, and *HAVCR2*, at the single-cell level. Correlation coefficients (R) and corresponding *p*-values were calculated.

### 2.10. Quantitative Real-Time PCR (q-PCR)

To determine the expression level of *NLRC5* in cells, total RNA was extracted from cells using TRIzol Reagent (Life Technologies, Carlsbad, CA, USA) according to the manufacturer’s instructions. Complementary DNA (cDNA) was synthesized using a reverse transcription kit (Takara, Japan). Quantitative PCR (qPCR) was performed on an Applied Biosystems StepOnePlus system using the SYBR Premix Ex Taq II Kit (Takara, Japan). Relative *NLRC5* mRNA expression was calculated using the 2^−ΔΔCt^ method with GAPDH as the internal reference. The primer sequences were as follows: Forward (F): 5′-CGACTTCTCAGGCAATGCTC-3′; Reverse (R): 5′-TCAGGAGGATGTGTTGGCTT-3′.

### 2.11. Definition of NLRC5 Expression Groups

Across all cohorts, *NLRC5* expression was stratified using two complementary approaches depending on the analytical purpose. For descriptive analyses, immune infiltration comparisons, methylation analysis and functional enrichment analyses, patients were dichotomized into *NLRC5*-high and *NLRC5*-low groups according to the median *NLRC5* expression within each cohort.

For survival analyses, including Kaplan–Meier analysis and Cox proportional hazards regression, the optimal cutoff value for *NLRC5* expression was determined using a data-driven method that maximized the separation of overall survival between groups. This approach was applied independently within each cohort to account for cohort-specific expression distributions.

### 2.12. Primary Human T Cells

Primary human T cells were derived from peripheral blood mononuclear cells (PBMCs) purchased from a commercial supplier. Human T cells were subsequently enriched using the EasySep Human T Cell Enrichment Kit (Stemcell Technologies, Vancouver, BC, Canada, Cat#19051) according to the manufacturer’s instructions.

Purified T cells were activated with anti-CD3/CD28 Dynabeads (Thermo Fisher Scientific, Waltham, MA, USA, Cat#11131D) at a bead-to-cell ratio of 1:1. Cells were maintained in X-VIVO 15 medium (Lonza) supplemented with 5% (*v*/*v*) heat-inactivated fetal bovine serum and 400 IU/mL recombinant human interleukin-2 (IL-2; Sino Biological Inc., Beijing, China, Cat#GMP-11848-HNAE).

### 2.13. Electroporation of CAR-T Cells

CRISPR/Cas9-mediated gene editing in primary human T cells was carried out via electroporation of Cas9/sgRNA ribonucleoprotein (RNP) complexes using the 4D-Nucleofector System N (Lonza) and the Primary Cell 4D-Nucleofector Kit (Lonza, Basel, Switzerland, Cat#V4XC-1032). Briefly, Cas9 protein (6 μg) (GenScript, Nanjing, China, Z03469-1) and sgRNA (6 μg) were incubated together at room temperature for 30 min to allow RNP complex formation.

CD3^+^ T cells were harvested 48 h after viral transduction, centrifuged at 300× *g* for 5 min, and resuspended in 20 μL of nucleofection buffer. The preassembled RNP complexes were diluted in an equal volume (20 μL) of the same buffer and mixed with the cell suspension. The mixture was transferred into a 16-well nucleocuvette strip and electroporated using the EO-115 program.

Following electroporation, cells were immediately transferred into 200 μL of pre-warmed T cell culture medium and expanded under standard conditions as described above. Gene disruption efficiency was assessed three days post-electroporation using Tracking of Indels by Decomposition (TIDE) analysis. Subsequent in vitro functional assays were performed seven days after electroporation.

The sgRNA sequences used for gene targeting were as follows:

*NLRC5*-sg1: atgtccagggttcggacacc; *NLRC5*-sg2: acaggttcttgttgccgagc; *NLRC5*-sg3: actgccaggtgtccgaaccc; *NLRC5*-sg4: tggaatccaggtccgtgttg; *NLRC5*-sg5: atccttagacactccggag.

### 2.14. Survival Analysis and Cox Regression Analysis

Survival analysis was performed to evaluate the association between *NLRC5* expression and overall survival (OS) in ESCC patients. Kaplan–Meier survival curves were generated for the *NLRC5*-high and *NLRC5*-low groups defined by the optimal cutoff value described above, and differences between groups were assessed using the log-rank test.

Univariate and multivariate Cox proportional hazards regression analyses were performed to assess the prognostic significance of *NLRC5* expression and clinicopathological variables. Hazard ratios (HRs) and 95% confidence intervals (CIs) were calculated to estimate the relative risk. Variables with potential prognostic relevance were included in the multivariate model to determine independent prognostic factors for overall survival.

## 3. Results

### 3.1. NLRC5 Is Overexpressed in ESCC and Predicts Poor Patient Survival

**Figure 1:**
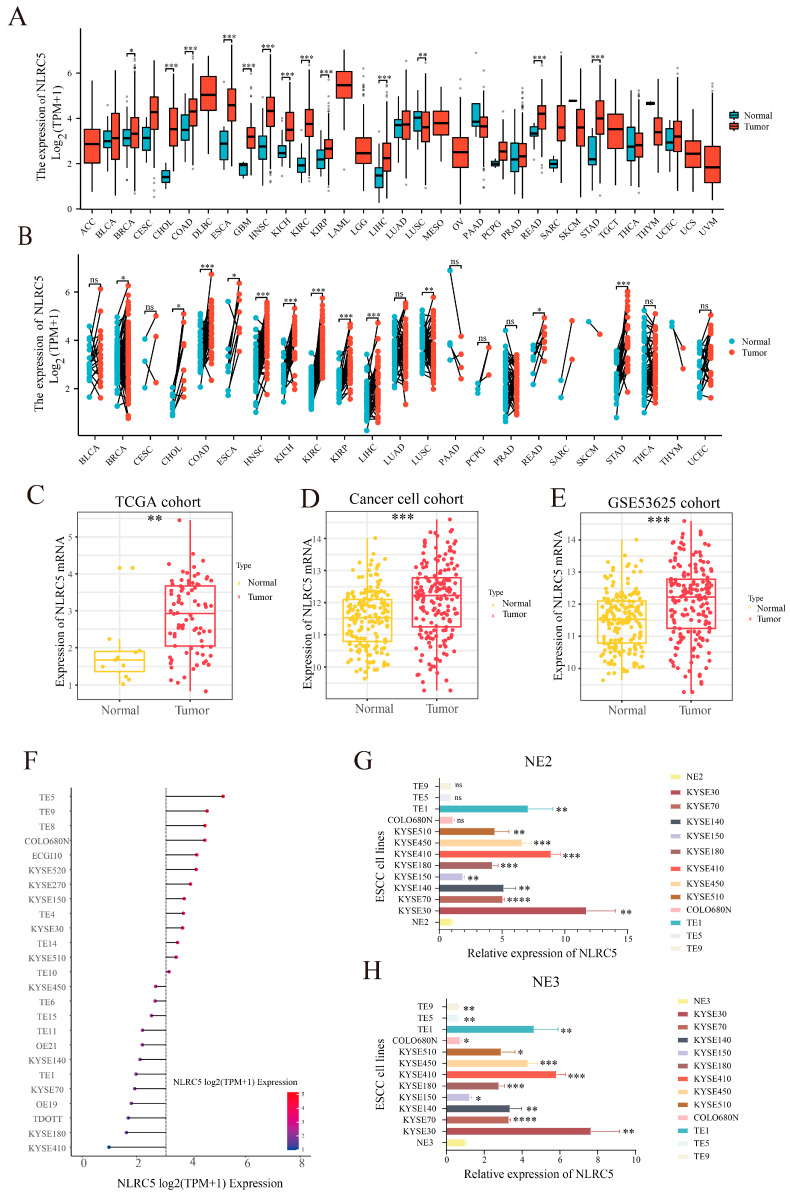
Pan-cancer analysis of *NLRC5* expression. (**A**) Box plot comparing *NLRC5* expression (log2 TPM + 1) between tumor and normal tissues across pan-cancer samples from the TCGA database. Each box represents the interquartile range (IQR), with the median indicated by a horizontal line. Whiskers extend to 1.5 × IQR, and dots represent outliers. (**B**) Paired dot plot showing *NLRC5* expression (log2 TPM + 1) in matched tumor and adjacent normal tissues from the TCGA database. Each line connects paired samples from the same patient. (**C**–**E**) Box plots illustrating NLRC5 mRNA expression in three independent ESCC cohorts. (**C**): TCGA cohort (normal, *n* = 11; tumor, *n* = 80); (**D**): CancerCell cohort (our cohort, normal, *n* = 155; tumor, *n* = 155); (**E**): GSE53625 cohort (normal, *n* = 179; tumor, *n* = 179). Statistical significance was assessed using an unpaired *t*-test. (**F**) Bar graph showing the expression of *NLRC5* in ESCC cell lines from the CCLE database. (**G**,**H**) qPCR results demonstrating the relative expression of *NLRC5* mRNA in ESCC cell lines compared to normal esophageal cells (NE2 (**G**) and NE3 (**H**)). Data are presented as mean ± SD from three independent experiments (*n* = 3). Statistical significance was assessed using an unpaired *t*-test (ns, *p* > 0.05; * *p* < 0.05; ** *p* < 0.01; *** *p* < 0.001).

To explore the clinical relevance of *NLRC5* in ESCC, we first assessed its expression in multiple cohorts. Analysis of pan-cancer TCGA data revealed elevated *NLRC5* transcript levels in tumor tissues compared to normal counterparts across most cancer types, including ESCA (esophageal carcinoma) (Figure 1A,B). This finding was validated in three independent ESCC cohorts, where *NLRC5* expression was significantly higher in tumor samples than in paired or unpaired normal esophageal tissues (Figure 1C–E and Figure S1A,B). We further examined *NLRC5* expression in ESCC cell lines using the data from the CCLE database, which showed variable expression across different lines (Figure 1F). To validate these findings in our experimental system, we performed qPCR analysis on available cell lines in our lab. This confirmed that the majority of ESCC cell lines exhibited higher *NLRC5* mRNA expression levels compared to immortalized normal esophageal epithelial cell lines NE2 and NE3, although COLO680N, TE5, and TE9 showed comparable levels to NE2 but lower than in NE3 (Figure 1G,H).

**Figure 2:**
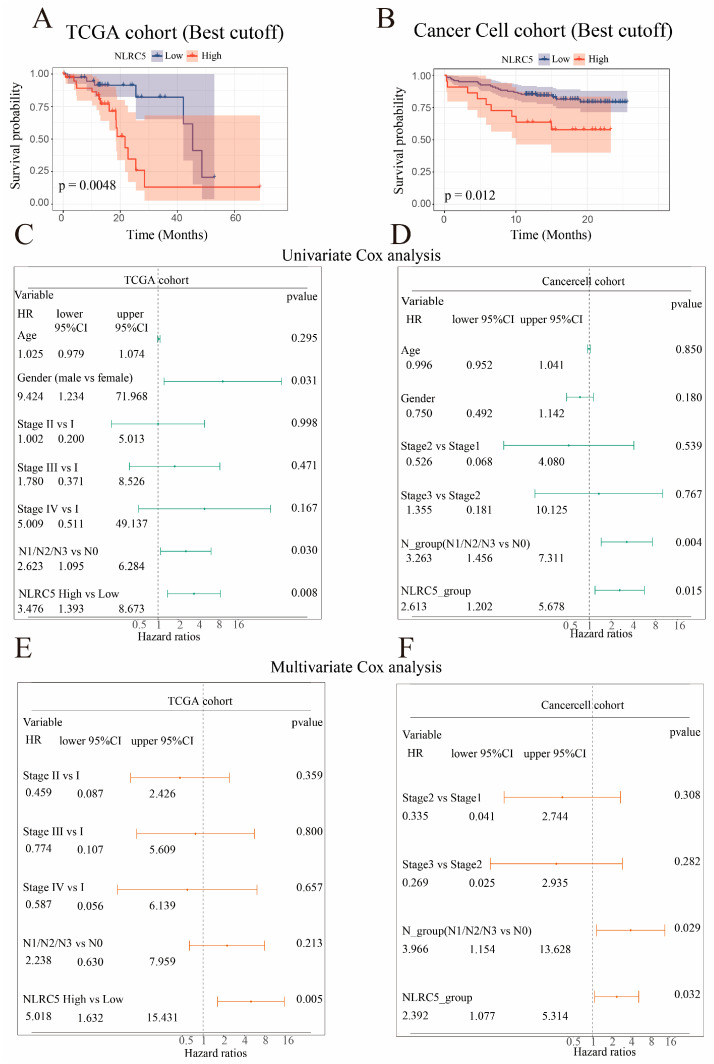
Prognostic significance of *NLRC5* expression in esophageal squamous cell carcinoma (ESCC) across different cohorts. (**A**,**B**) Kaplan–Meier curves showing overall survival (OS) of ESCC patients with high or low *NLRC5* expression in the (**A**) TCGA cohort and (**B**) CancerCell cohort. Patients were stratified according to the optimal cut-off determined by the log-rank test. *p* values were calculated using the log-rank test. (**C**,**D**) Univariate Cox regression analysis of OS in the (**C**) TCGA cohort and (**D**) CancerCell cohort. Hazard ratios (HRs), 95% confidence intervals (CIs), and *p* values are shown for each variable. (**E**,**F**) Multivariate Cox regression analysis of OS in the (**E**) TCGA cohort and (**F**) CancerCell cohort after adjustment for clinicopathological factors. HRs, 95% CIs, and *p* values are presented for each factor.

We next investigated the prognostic significance of *NLRC5*. Kaplan–Meier survival analysis demonstrated that patients with high NLRC5 expression had significantly poorer overall survival (OS) in both the TCGA (*p* = 0.0048) and CancerCell (*p* = 0.012) cohorts (Figure 2A,B). Univariate Cox regression confirmed NLRC5 expression as a significant prognostic factor for mortality in both the TCGA (HR = 3.476, 95% CI = 1.393–8.673, *p* = 0.008) and CancerCell cohorts (HR = 2.613, 95% CI = 1.202–5.678, *p* = 0.015) (Figure 2C,D). Crucially, multivariate analysis adjusting for clinicopathological variables identified high *NLRC5* expression as an independent prognostic factor for poor OS in both the TCGA (HR = 5.018, 95% CI = 1.632–15.431, *p* = 0.005) and CancerCell cohorts (HR = 2.392, 95% CI = 1.077–5.314, *p* = 0.032) (Figure 2E,F). Together, these results demonstrate that elevated *NLRC5* expression is independently associated with unfavorable prognosis in patients with ESCC.

### 3.2. NLRC5 Hypomethylation Contributes to Its Upregulation in ESCC

**Figure 3:**
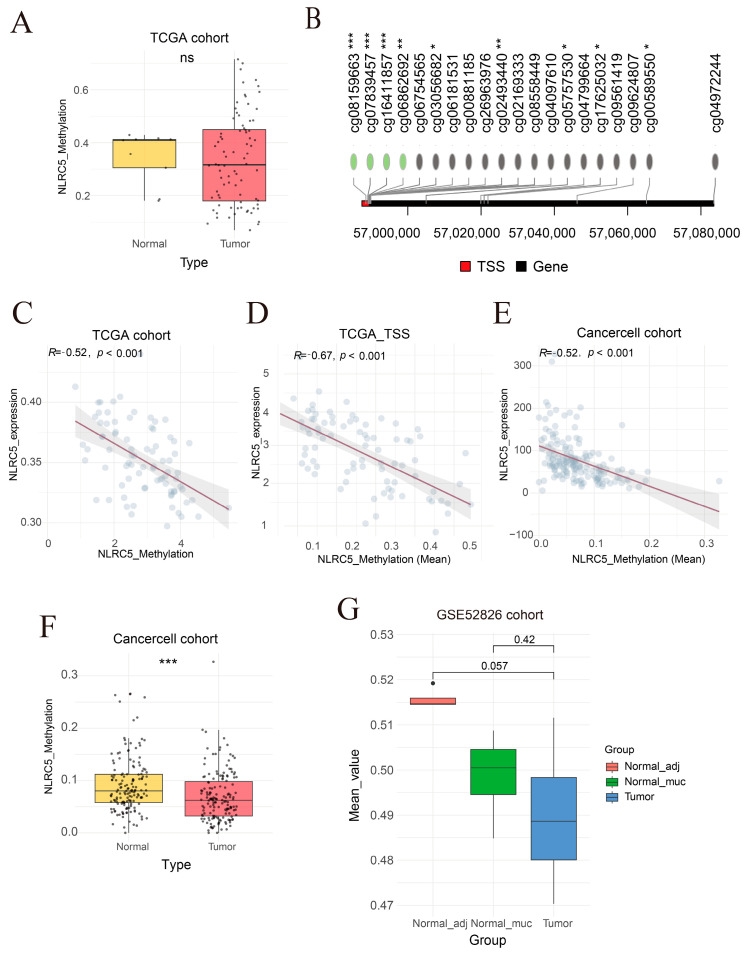
The relationship between *NLRC5* methylation levels and its mRNA expression in various cohorts. (**A**) Box plot showing the average methylation levels of *NLRC5* in tumor versus normal tissues in the TCGA cohort, analyzed using a non-paired *t*-test (normal, *n* = 11; tumor, *n* = 80). (**B**) Lollipop plot showing the relationship between *NLRC5* methylation and gene expression in ESCC. Red dots represent transcription start sites (TSS), while green dots indicate correlation coefficients (R) greater than 0.3. (**C**) Scatter plot displaying the Spearman correlation analysis between *NLRC5* mRNA expression and the average methylation levels of *NLRC5*. *p* values were adjusted using the Benjamini–Hochberg false discovery rate (FDR) method. (**D**) Scatter plot showing the Spearman correlation between *NLRC5* mRNA expression and the average methylation levels in the TSS region of *NLRC5*. (**E**) Scatter plot representing the Spearman correlation analysis between *NLRC5* mRNA expression and the average methylation levels of *NLRC5* across the entire gene. (**F**) Box plot comparing the average methylation levels of *NLRC5* in tumor versus normal tissues in the Cancer Cell cohort, analyzed using a non-paired *t*-test (normal, *n* = 155; tumor, *n* = 155). (**G**) Box plot comparing the average methylation levels of *NLRC5* in tumor, adjacent normal tissue, and normal muscle in the GSE52826 cohort, analyzed using a non-paired *t*-test (tumor, *n* = 4; adjacent normal, *n* = 4; normal mucosa, *n* = 4) (ns, *p* > 0.05, *, *p* < 0.05, **, *p* < 0.01, ***, *p* < 0.001).

To investigate the mechanism underlying *NLRC5* upregulation, we examined its epigenetic regulation through DNA methylation analysis. DNA methylation data from three independent cohorts (TCGA, CancerCell, GSE52826) were analyzed (TCGA, CancerCell, and GSE52826) (Figure 3A,F,G). In the GSE52826 cohort, *NLRC5* methylation levels tended to be lower in tumor tissues compared with normal tissues (*p* = 0.057), suggesting a potential association between reduced methylation and *NLRC5* upregulation in ESCC.

Furthermore, Spearman correlation analysis demonstrated a significant inverse relationship between *NLRC5* methylation and its mRNA expression across all cohorts, particularly at sites near the transcription start site (Figure 3B–E). These results suggest that *NLRC5* overexpression in ESCC is at least partially driven by the loss of promoter methylation.

### 3.3. NLRC5 Expression Is Strongly Associated with Immune-Related Transcriptional Programs

**Figure 4:**
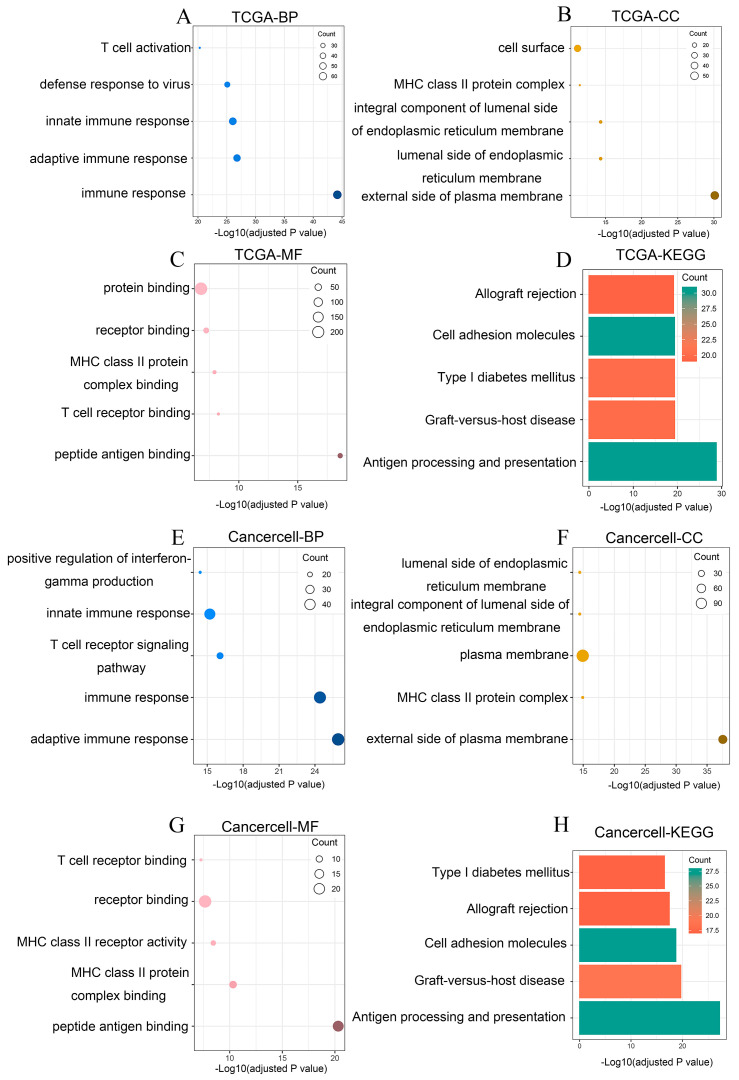
Functional enrichment analysis of genes correlated with *NLRC5* in TCGA and CancerCell cohorts. (**A**–**D**) represent the TCGA cohort: (**A**) Gene Ontology (GO) biological process (BP) analysis showing significant enrichment in immune-related processes such as T cell activation, defense response to virus, and adaptive immune response. (**B**) GO cellular component (CC) analysis highlighting enrichment in cell surface and various membrane-related components. (**C**) GO molecular function (MF) analysis showing enrichment in protein binding, receptor binding, and MHC class II protein complex binding. (**D**) KEGG pathway analysis indicating significant enrichment in pathways related to allograft rejection, cell adhesion molecules, type I diabetes mellitus, and antigen processing and presentation. (**E**–**H**) represent the CancerCell cohort: (**E**) GO biological process (BP) analysis showing significant enrichment in pathways related to the immune response, T cell receptor signaling, and interferon-gamma production. (**F**) GO cellular component (CC) analysis highlighting enrichment in various membrane-related components similar to the TCGA cohort. (**G**) GO molecular function (MF) analysis indicating significant enrichment in receptor binding, MHC class II receptor activity, and T cell receptor binding. (**H**) KEGG pathway analysis showing significant enrichment in pathways related to type I diabetes mellitus, allograft rejection, and antigen processing and presentation. (The size of each bubble corresponds to the number of genes enriched in the respective pathway. Bubble color reflects the statistical significance of enrichment, represented as −log10(FDR), with darker blue indicating more significant enrichment).

Given the established role of *NLRC5* in immune regulation, we performed functional enrichment analysis on genes significantly correlated with *NLRC5* expression in the TCGA and CancerCell cohorts (|R| > 0.5, *p* < 0.05). In both the TCGA and CancerCell cohorts as well as GSE53625, Gene Ontology (GO) analysis revealed robust enrichment for biological processes related to immune activation, including “T cell activation,” “adaptive immune response,” and “antigen processing and presentation” (Figure 4A–C,E–G). Correspondingly, the analysis highlighted enrichment for “Antigen processing and presentation,” “Graft-versus-host disease,” and “Cell adhesion molecules” (Figure 4D,H). These consistent findings across independent datasets confirm that *NLRC5* expression in ESCC is tightly linked to broader immune response pathways, particularly those governing T-cell activity and antigen presentation (Figure S2).

### 3.4. High NLRC5 Expression Defines an Immune-Inflamed but Functionally Impaired TME

**Figure 5:**
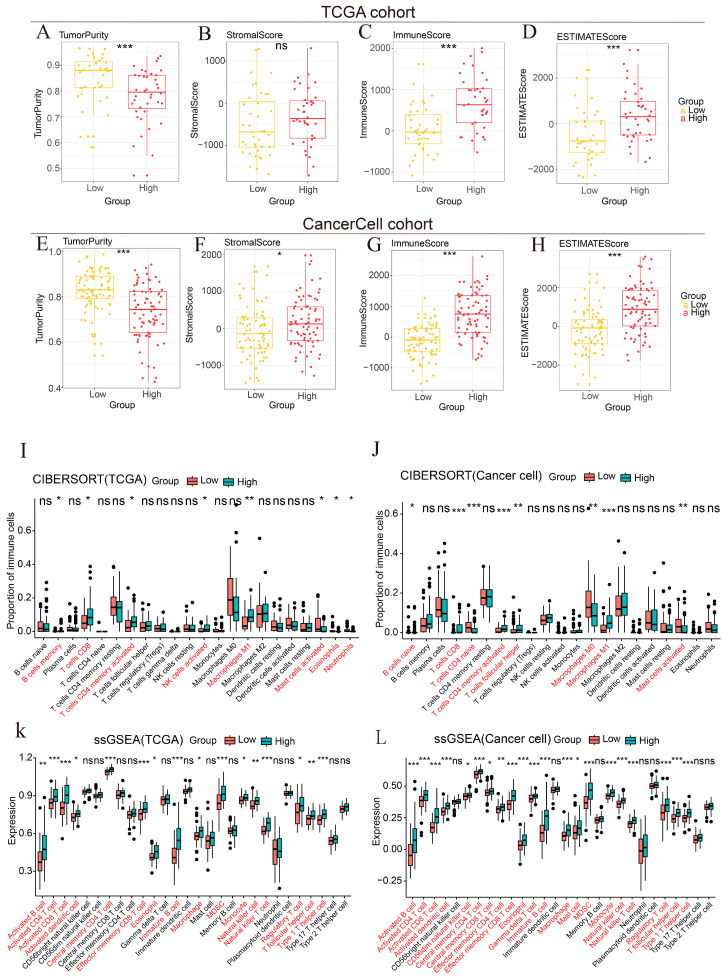
Immune infiltration characteristics associated with NLRC5 expression in the TCGA and CancerCell cohorts. (**A**–**D**) Box plots representing Tumor Purity, Stromal Score, Immune Score, and ESTIMATE Score in the TCGA cohort (*n* = 80), stratified by *NLRC5* expression levels (Low vs. High). Statistical comparisons were performed using unpaired Student’s *t*-tests. (**E**–**H**) Corresponding box plots for the CancerCell cohort (*n* = 155), evaluating the same immune-related scores. The consistency between the two cohorts underscores the robustness of *NLRC5* as a potential immune modulator. (**I**,**J**) CIBERSORT analysis showing the relative proportions of various immune cell types between *NLRC5* low and high expression groups in the TCGA (*n* = 80) and CancerCell (*n* = 155) cohorts. The analysis reveals significant variations in immune cell infiltration, particularly in subsets such as T cells and macrophages, where high *NLRC5* expression correlates with increased immune cell presence. (**K**,**L**) ssGSEA (single-sample Gene Set Enrichment Analysis) showing enrichment patterns of immune-related gene signatures in NLRC5-high versus NLRC5-low tumors in the TCGA and CancerCell cohorts. Elevated NLRC5 expression was associated with increased activation of multiple immune-related pathways (ns, *p* > 0.05, * *p* < 0.05, ** *p* < 0.01, *** *p* < 0.001).

The association of *NLRC5* with both poor prognosis and immune activation presented a paradox. We hypothesized that while *NLRC5* might promote immune cell infiltration, it could also be linked to a dysfunctional state of these infiltrating cells. To test this, we first applied the ESTIMATE algorithm, which showed that high *NLRC5* expression was associated with significantly higher Immune, Stromal, and ESTIMATE scores, and lower tumor purity in both the TCGA and CancerCell cohorts (Figure 5A–H). This indicates that *NLRC5*-high tumors are indeed more immune-infiltrated. Deconvolution of the immune infiltrate using CIBERSORT and ssGSEA confirmed this, revealing a significant enrichment of multiple immune cell types, including CD8^+^ T cells, activated CD4^+^ T cells, and NK cells, in the *NLRC5*-high group (Figure 5I–L). Consistent results were observed in the independent GSE53625 cohort using CIBERSORT and ssGSEA (Figure S3). In addition, analysis using the xCell algorithm showed similar immune infiltration patterns (Figure S4).

**Figure 6:**
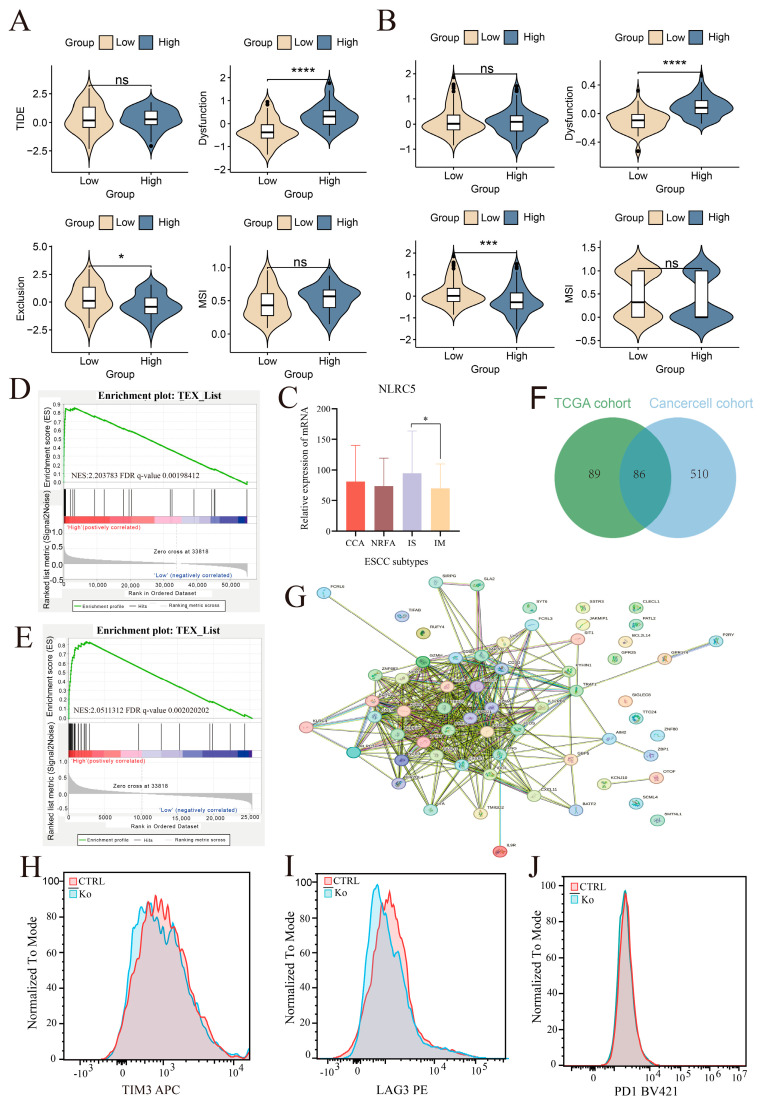
*NLRC5* high expression is associated with immune escape and T cell exhaustion pathways in ESCC. (**A**,**B**) Violin plots showing Tumor Immune Dysfunction and Exclusion (TIDE) analysis in the TCGA cohort (**A**) and CancerCell cohort (**B**). Tumors with high *NLRC5* expression exhibited higher T-cell dysfunction scores and immune escape-related signatures compared with the *NLRC5*-low group. (**C**) *NLRC5* expression across four ESCC molecular subtypes in the CancerCell cohort. Tumors were classified into four subtypes as previously reported: CCA (cell cycle activation, *n* = 39), NRFA (NRF2 oncogenic activation, *n* = 38), IS (immune suppression, *n* = 30), and IM (immune modulation, *n* = 48). *NLRC5* expression was significantly higher in the IS subtype compared with the IM subtype, suggesting a potential association between *NLRC5* expression and an immunosuppressive tumor microenvironment. (**D**,**E**) Gene Set Enrichment Analysis (GSEA) in the TCGA (**D**) and CancerCell (**E**) cohorts, showing significant upregulation of T cell exhaustion pathways in the *NLRC5* high expression group. (**F**) Venn diagram illustrating overlapping differentially expressed genes (DEGs) between NLRC5 high and low expression groups in the TCGA and CancerCell cohorts. (**G**) A protein–protein interaction (PPI) network constructed from the overlapping DEGs, highlighting the key gene interactions potentially driven by NLRC5 expression, with several immune-related genes showing strong connectivity. (**H**–**J**) Representative flow cytometry histograms showing the expression of exhaustion markers TIM-3 (**H**), LAG-3 (**I**), and PD-1 (**J**) in control (CTRL) and NLRC5 knockout (KO) primary human T cells following co-culture with tumor cells. The y-axis indicates normalized cell counts, and the x-axis represents mean fluorescence intensity (MFI). Data shown are representative of three independent biological replicates (*n* = 3) (ns, *p* > 0.05, * *p* < 0.05, *** *p* < 0.001, **** *p* < 0.0001).

To assess the functional state of these infiltrating T cells, we employed the TIDE (Tumor Immune Dysfunction and Exclusion) framework. While the overall TIDE score was not consistently different, the “Dysfunction” score—which specifically predicts T-cell dysfunction in tumors with high cytotoxic T lymphocyte (CTL) infiltration—was significantly elevated in the *NLRC5*-high group of both cohorts (Figure 6A,B). This suggests that the abundant CD8^+^ T cells in *NLRC5*-high tumors may be in a dysfunctional or exhausted state. Consistent with this observation, *NLRC5* expression was positively correlated with multiple immune checkpoint molecules, including *PDCD1* (PD-1), *CD274* (PD-L1), *CTLA4*, *HAVCR2* (TIM-3), and *LAG3*, in both the TCGA and CancerCell cohorts. Furthermore, these checkpoint genes were significantly upregulated in the NLRC5-high group (Figure S6A–D), further supporting the presence of an immunosuppressive immune context associated with elevated *NLRC5* expression.

To further explore the relationship between *NLRC5* expression and the immune landscape of ESCC, we analyzed *NLRC5* expression across the four molecular subtypes defined in the Cancer Cell cohort. *NLRC5* expression was not uniformly distributed across the subtypes and was significantly higher in the immunosuppressive (IS) subtype compared with the immune-modulated (IM) subtype (Figure 6C). Given that the IM subtype is characterized by a more active immune microenvironment, this result further suggests that elevated *NLRC5* expression may be associated with an immunosuppressive immune state.

This was corroborated by GSEA, which showed significant enrichment of a T-cell exhaustion gene signature in the *NLRC5*-high groups (Figure 6D,E). Furthermore, analysis of overlapping differentially expressed genes between the *NLRC5*-high and -low groups from both cohorts revealed a protein–protein interaction network (PPI) centered on key immune checkpoint molecules such as PD-1, CTLA-4, and TIM-3 (Figure 6F,G).

Finally, to provide functional evidence, we generated NLRC5- knockout (KO) primary human T cells with CRISPR/Cas9. TIDE analysis was performed to determine the indel efficiency (Figure S5J), and the knockout efficiency of NLRC5 was further validated by qPCR and Western blotting (Figure S6E,F). Upon co-culture with tumor cells, NLRC5-KO T cells displayed a marked reduction in the expression of exhaustion markers TIM-3 and LAG-3 compared to control T cells (Figure 6H–J). Collectively, these data demonstrate that while *NLRC5* expression is associated with a highly inflamed TME, it is also linked to the establishment of T-cell exhaustion, which may explain its paradoxical association with poor survival.

### 3.5. Single-Cell Analysis Confirms NLRC5 Expression in T Cells and Its Correlation with Exhaustion Markers

**Figure 7:**
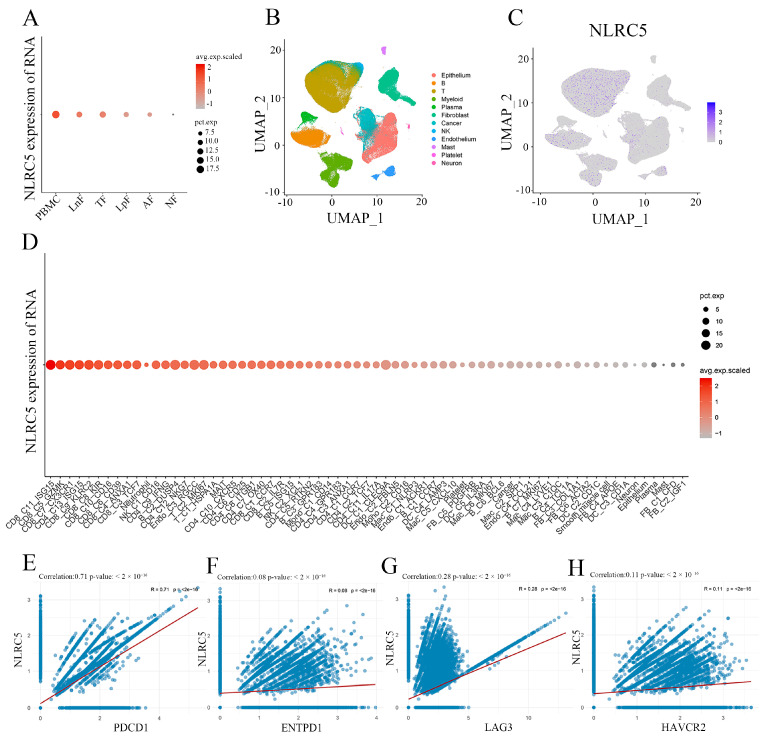
The expression and correlation of *NLRC5* across different tissues and immune cell subtypes in single-cell RNA sequencing (scRNA-seq) data. (**A**) Dot plot showing *NLRC5* RNA expression across various tissue types, including peripheral blood mononuclear cells (PBMCs), lymph nodes (normal and metastatic), and esophageal tissues. Notably, *NLRC5* is highly expressed in PBMC, suggesting its significant involvement in circulating immune cells. (**B**) UMAP plot of T cell subtypes from scRNA-seq data, highlighting various immune subpopulations including CD8^+^ T cells, Tregs, and other immune cells. (**C**) UMAP plot depicting *NLRC5* expression levels across T cell subtypes, with notable enrichment in certain immune cell populations, indicating its role in immune modulation. (**D**) Dot plot illustrating the expression levels of *NLRC5* in various immune cell subtypes, particularly high in T cells and certain myeloid cells. This pattern further emphasizes *NLRC5*’s involvement in immune processes within the tumor microenvironment. (**E**–**H**) Correlation analysis of *NLRC5* with immune checkpoint molecules within the CD8^+^ T cell subset. Strong positive correlations are observed between *NLRC5* and key immune checkpoint markers, including *PDCD1* (**E**) (R = 0.71, *p* < 2 × 10^−16^), ENTPD1 (**F**) (R = 0.08, *p* < 2 × 10^−16^), LAG3 (**G**) (R = 0.28, *p* < 2 × 10^−16^), and *HAVCR2* (**H**) (R = 0.11, *p* < 2 × 10^−16^). These correlations suggest *NLRC5* may influence immune checkpoint pathways and contribute to T cell exhaustion within the tumor microenvironment. The red line indicates the fitted linear regression line.

To refine our understanding of *NLRC5*’s role within the TME, we turned to single-cell RNA sequencing (scRNA-seq). UMAP projection of immune cells from ESCC tumors revealed distinct clusters of myeloid and lymphoid lineages (Figure 7A,B). *NLRC5* expression was detected across multiple immune subsets but was most prominently enriched in CD8^+^ T cells and, to a lesser extent, in myeloid cells (Figure 7C,D). This expression pattern supports a direct role for *NLRC5* in T-cell biology within the tumor.

We then examined the relationship between *NLRC5* expression and immune checkpoint molecules specifically within the CD8^+^ T-cell population. Strikingly, we observed significant positive correlations between *NLRC5* and several exhaustion-associated checkpoints, including *PDCD1* (PD-1, R = 0.71, *p* < 2 × 10^−16^) and *LAG3* (R = 0.28, *p* < 2 × 10^−16^) (Figure 7E–H). These single-cell level data provide high-resolution evidence linking *NLRC5* expression in CD8^+^ tumor-infiltrating lymphocytes with a transcriptional program of T-cell exhaustion, further solidifying the findings from our bulk analyses.

### 3.6. NLRC5 Expression Is Associated with Transcriptional Signatures of PANoptosis

**Figure 8:**
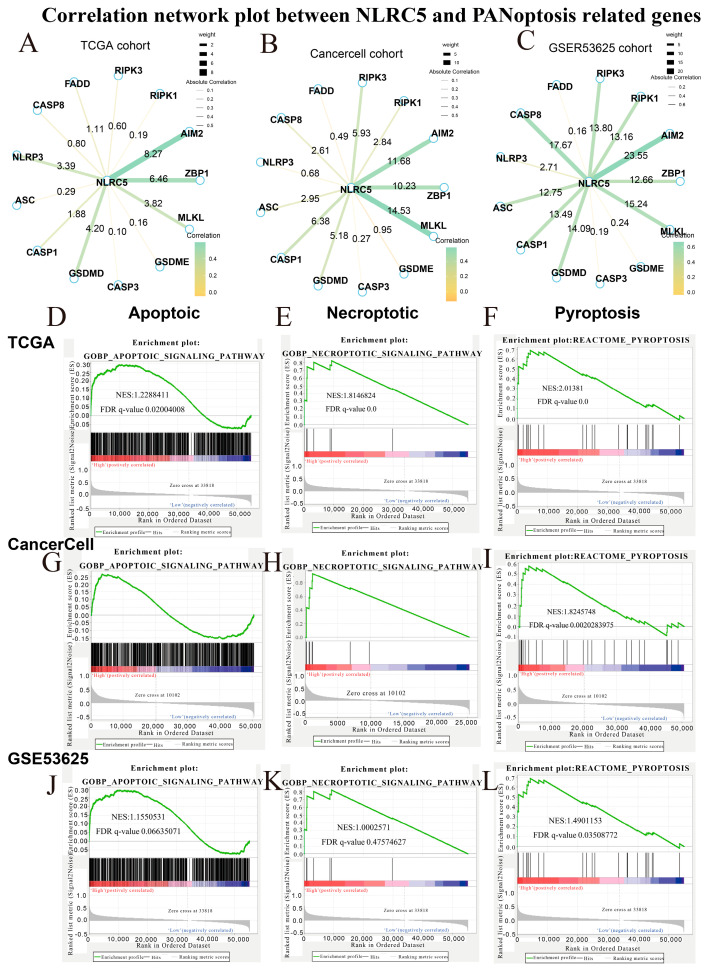
Correlation network and pathway enrichment analysis of *NLRC5* and PANoptosis-related genes across three cohorts. (**A**–**C**) Correlation network of *NLRC5* with PANoptosis-related genes in the TCGA cohort (**A**), CancerCell cohort (**B**), and GSE53625 cohort (**C**). The strength of the correlations is depicted by the thickness of the lines, while the direction and significance of the correlations are represented by color shading. (**D**–**F**) GSEA (Gene Set Enrichment Analysis) of *NLRC5* expression in the TCGA cohort, comparing high and low *NLRC5* expression groups. Enriched pathways include (**D**) apoptotic signaling, (**E**) necroptotic signaling, and (**F**) pyroptosis pathways. NES (Normalized Enrichment Score) and FDR (False Discovery Rate) q-values are indicated for each pathway. (**G**–**I**) GSEA in the CancerCell cohort, showing enrichment in the apoptotic (**G**), necroptotic (**H**), and pyroptotic (**I**) pathways. (**J**–**L**) GSEA in the GSE53625 cohort, illustrating similar enrichment patterns in the apoptotic (**J**), necroptotic (**K**), and pyroptotic (**L**) pathways. NES and FDR values are shown for each cohort and pathway.

Given the multifaceted role of *NLRC5* in cell death pathways, we next investigated whether its expression might be linked to programmed cell death of immune cells, potentially representing an additional immunosuppressive mechanism. We observed a strong and consistent positive correlation between *NLRC5* and several key regulators of PANoptosis—an integrated cell death pathway encompassing pyroptosis, apoptosis, and necroptosis—across all three bulk cohorts. Notably, *AIM2* and *GSDME* showed the most robust correlations (Figure 8A–C). To test this association more directly, we performed GSEA for the individual cell death pathways. Tumors with high *NLRC5* expression were significantly enriched for gene signatures of apoptosis, necroptosis, and pyroptosis in both the TCGA and CancerCell cohorts (Figure 8D–I). A similar trend was observed in the GSE53625 cohort, with significant enrichment for apoptosis and pyroptosis (Figure 8J–L). These results suggest that beyond promoting T-cell exhaustion, a high *NLRC5* expression state is also characterized by transcriptional programs associated with PANoptosis, pointing to a multi-faceted role in sculpting an immunosuppressive TME.

## 4. Discussion

In this study, we provide the first systematic characterization of *NLRC5* within the ESCC TME. Through multi-cohort transcriptomic, epigenomic, and single-cell analyses, we demonstrate that *NLRC5* expression is associated with poor prognosis and features of an immunosuppressive tumor microenvironment. Notably, elevated *NLRC5* expression is associated with a tumor microenvironment that displays both immune-inflamed and functionally suppressed characteristics, including exhausted CD8^+^ T cells and transcriptional signatures related to PANoptosis.

The paradox of high CD8^+^ T cell infiltration coupled with poor clinical outcome is resolved by our data showing a strong association between *NLRC5* and T-cell exhaustion. The elevated T-cell dysfunction score, the enrichment of exhaustion gene signatures, and the striking correlation between *NLRC5* and checkpoint molecules like *PDCD1* at the single-cell level all point to *NLRC5* as a potential driver of this dysfunctional state. Our preliminary functional data, showing that *NLRC5* knockout in primary human T cells reduces exhaustion marker expression, provides a causal link and suggests that *NLRC5* itself may contribute to the maintenance of an exhausted phenotype. This positions *NLRC5* as more than a passive marker; it may act as an “immunoregulatory hub” that orchestrates the transition from an effective anti-tumor response to a state of immune paralysis. These findings align with and extend a growing body of literature that implicates *NLRC5* in immune evasion in specific contexts, moving beyond its canonical role in antigen presentation [24,25,26].

Our investigation further uncovered an association between *NLRC5* and PANoptosis. The correlation with key PANoptosis regulators and the consistent enrichment of apoptosis, necroptosis, and pyroptosis pathways in *NLRC5*-high tumors suggest an additional layer of immune suppression. We hypothesize that within the metabolically stressed ESCC TME, *NLRC5* may act as an immunometabolic checkpoint, integrating cues to dictate the fate of infiltrating immune cells—steering them towards either functional exhaustion or programmed cell death [10,27,28,29]. This hypothesis provides a compelling framework for future studies to explore the crosstalk between innate immunity, metabolism, and cell death in ESCC.

The strength of our study lies in its rigorous multi-omics approach, integrating data from over 400 patient samples and employing a suite of complementary computational tools to dissect the TME at multiple levels. However, we acknowledge its primary limitation: the correlative nature of the findings. While our bioinformatic analyses and preliminary in vitro experiments are highly suggestive, direct causal relationships between *NLRC5* and the proposed mechanisms require further validation through loss- and gain-of-function studies in vivo. Future work should focus on elucidating the precise molecular pathways by which *NLRC5* promotes T-cell exhaustion and PANoptosis, and on exploring whether its therapeutic targeting can synergize with existing immunotherapies, such as immune checkpoint blockade or CAR-T cells [9,30,31], to enhance anti-tumor efficacy in ESCC.

In conclusion, our comprehensive analysis establishes *NLRC5* as a key negative regulator of anti-tumor immunity in ESCC. Its expression is characterized by a TME that is both highly inflamed and profoundly suppressed, potentially through the dual mechanisms of T-cell exhaustion and immune cell PANoptosis. While the precise molecular mechanisms remain to be fully elucidated, *NLRC5* emerges as a powerful prognostic biomarker and a compelling novel target for immunotherapeutic intervention in this deadly disease.

## 5. Conclusions

In summary, our study provides a comprehensive multi-omics characterization of *NLRC5* in ESCC and identifies it as a key regulator of the tumor immune microenvironment. Elevated *NLRC5* expression is associated with poor patient survival and a paradoxical immune contexture characterized by increased CD8^+^ T-cell infiltration accompanied by functional exhaustion and enhanced immune checkpoint signaling. In addition, *NLRC5*-high tumors exhibit enrichment of transcriptional programs related to PANoptosis, suggesting a potential contribution to immune cell dysfunction and loss within the tumor microenvironment. Collectively, these findings suggest that *NLRC5* may serve as a potential prognostic biomarker and provide insights into immune regulation in ESCC. Targeting *NLRC5* may represent a promising strategy to restore effective anti-tumor immunity and improve immunotherapeutic outcomes in ESCC.

## Data Availability

The publicly available datasets used in this study can be accessed from The Cancer Genome Atlas (TCGA; https://portal.gdc.cancer.gov/) and the Gene Expression Omnibus (GEO; GSE53625 and GSE52826). The in-house ESCC cohort has been previously described and published (DOI: 10.1016/j.ccell.2022.12.004).

## Supplementary Materials

The following supporting information can be downloaded at: https://www.mdpi.com/article/10.3390/cancers18071117/s1, Figure S1. Association between NLRC5 and clinicopathological characteristics of ESCC. (A,B) Association between NLRC5 and clinicopathological characteristics of ESCC. (A) The landscape of NLRC5-related clinicopathological features of ESCC in the TCGA cohort; (B) The landscape of NLRC5-related clinicopathological features of ESCC in the CancerCell cohort. Figure S2: Immunological and functional enrichment analysis of the GSE53625 cohort. GO enrichment analysis of Biological Process (BP) (A), Cellular Component (CC) (B), and Molecular Function (MF) (C) categories in the GSE53625 cohort, with significant terms displayed based on their −log10 (adjust-p). BP terms, including mitochondrial translation, response to virus, and apoptotic process (A). CC terms, including cytosol, nucleoplasm, and mitochondrial inner membrane (B). MF terms, including protein binding, RNA binding, and ubiquitin-protein ligase binding (C). KEGG pathway analysis highlights the most enriched signaling pathways in the GSE53625 cohort, such as the Cytosolic DNA-sensing pathway and Epstein–Barr virus infection pathway (D). The x-axis indicates −log10 (FDR) values based on Benjamini–Hochberg adjusted *p* values, and the y-axis lists the enriched terms. Boxplots showing StromalScore (E), TumorPurity (F), StromalScore (G), and ImmuneScore (H) between high and low NLRC5 expression groups. Significant differences are indicated, and high expression groups demonstrate distinct immune profiles. Figure S3: Comparison of immune cell proportions between NLRC5-high and NLRC5-low expression groups in the GSE53625 cohort. CIBERSORT analysis showing the proportion of various immune cell types between NLRC5-high (cyan) and NLRC5-low (red) groups (A). Significant differences in immune cell infiltration are observed for multiple cell types, including increased levels of activated CD8^+^ T cells, M1 macrophages, and T follicular helper cells in the NLRC5-high group. ssGSEA (single-sample Gene Set Enrichment Analysis) results showing the expression of immune-related gene sets (B). NLRC5-high tumors display a significant enrichment of immune cell populations such as activated CD8^+^ T cells, activated dendritic cells, and M1 macrophages, reinforcing the role of NLRC5 in modulating the immune microenvironment of ESCC. Figure S4: Using the Xcell immune analysis method, the differences in immune cell abundance between high and low NLRC5 expression groups were analyzed in three cohorts: TCGA, Cancer Cell, and GSE53625. (A) represents the TCGA cohort (*n* = 80), (B) represents the Cancer Cell cohort (*n* = 155), and (C) represents the GSE53625 cohort (*n* = 179). Figure S5: Correlation between NLRC5 expression and T cell-related transcriptional signatures. (A) Effector T cell signatures showed a weak and non-significant correlation with NLRC5 expression. (B) Effector regulatory T (Treg) cells exhibited a moderate positive correlation with NLRC5 expression. (C) Exhausted T cell signatures demonstrated a strong positive correlation with NLRC5 expression. (D) Naive T cell signatures displayed a weak but statistically significant positive correlation with NLRC5 expression. (E) Resident T cell signatures were positively correlated with NLRC5 expression. (F) Resting Treg signatures showed a significant moderate positive correlation with NLRC5 expression. (G) Central memory T (Tcm) cell signatures were moderately correlated with NLRC5 expression. (H) Effector memory T (Tem) cell signatures exhibited a moderate positive correlation with NLRC5 expression. (I) Th1-like cell signatures showed a strong positive correlation with NLRC5 expression. (J) TIDE analysis of NLRC5 gene editing efficiency in primary human T cells. The x-axis indicates the size of insertions or deletions relative to the wild-type sequence, while the y-axis represents the percentage of total sequencing reads. Indel frequencies at the NLRC5 locus following CRISPR/Cas9 editing were quantified by TIDE analysis. Figure S6 Association between NLRC5 and immune checkpoint molecules and validation of NLRC5 knockout in T cells. (A,B) Radar plots showing the correlation between NLRC5 expression and immune checkpoint-related genes (PDCD1, CD274, CTLA4, CD80, CD86, LAG3, HAVCR2, and TIGIT) in the TCGA cohort (A) and the CancerCell cohort (B). Blue dashed lines indicate Pearson correlation coefficients (R), and red points indicate the corresponding *p*-values. (C,D) Expression levels of immune checkpoint-related genes in the NLRC5 high and low expression groups in the TCGA cohort (C) and CancerCell cohort (D). Gene expression differences between groups were compared using the Wilcoxon rank-sum test. Data are shown as boxplots indicating median and interquartile range. (E) Quantitative PCR analysis showing relative NLRC5 mRNA expression in control (CTRL) and CRISPR/Cas9-mediated NLRC5 knockout (KO) primary human T cells using three independent sgRNAs (ko1–ko3). (F) Western blot analysis confirming NLRC5 protein depletion in NLRC5-KO T cells compared with CTRL cells.

## Abbreviations

The following abbreviations are used in this manuscript:

ESCC	esophageal squamous cell carcinoma
TME	tumor immune microenvironment
CTLs	cytotoxic T lymphocytes
PBMCs	peripheral blood mononuclear cells
PPI	protein–protein interaction
KO	knockout
