# Supplementary material for: High NLRC5 Expression Is Associated with an Immunosuppressive Tumor Microenvironment and Poor Prognosis in Esophageal Squamous Cell Carcinoma
Source: Cancers (Basel). 2026 Mar 30;18(7):1117. doi: 10.3390/cancers18071117 (PMC13072022; doi:10.3390/cancers18071117)
Supplement: Supplementary file 1 [file cancers-18-01117-s001.zip › cancers-4199304-supplementary.pdf]

Supplementary Material

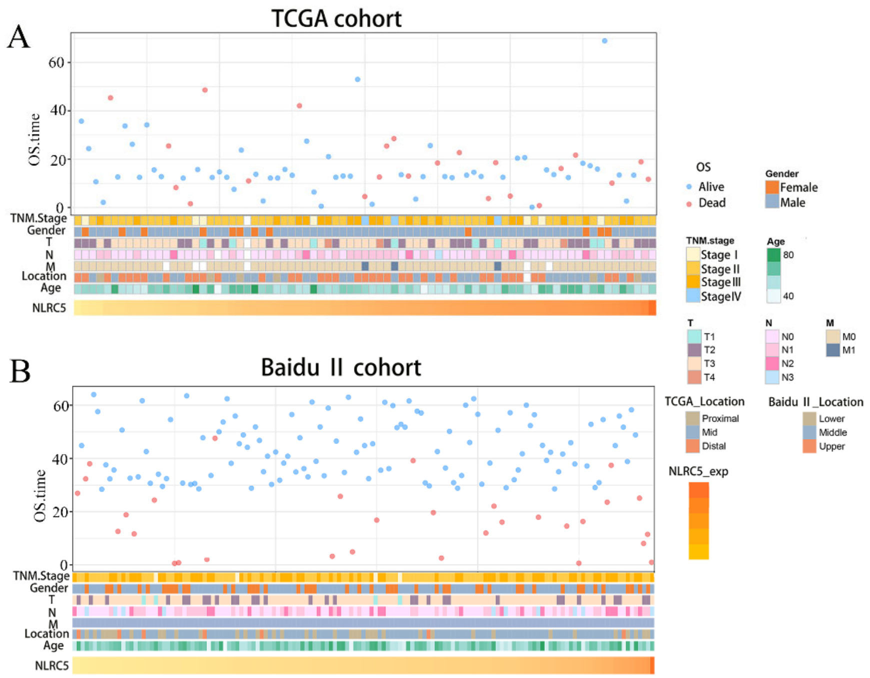

Figure S1. Association between *NLRC5* and clinicopathological characteristics of ESCC. (A-B) Association between *NLRC5* and clinicopathological characteristics of ESCC. (A) The landscape of *NLRC5*-related clinicopathological features of ESCC in the TCGA cohort;(B) The landscape of *NLRC5*-related clinicopathological features of ESCC in the CancerCell cohort.

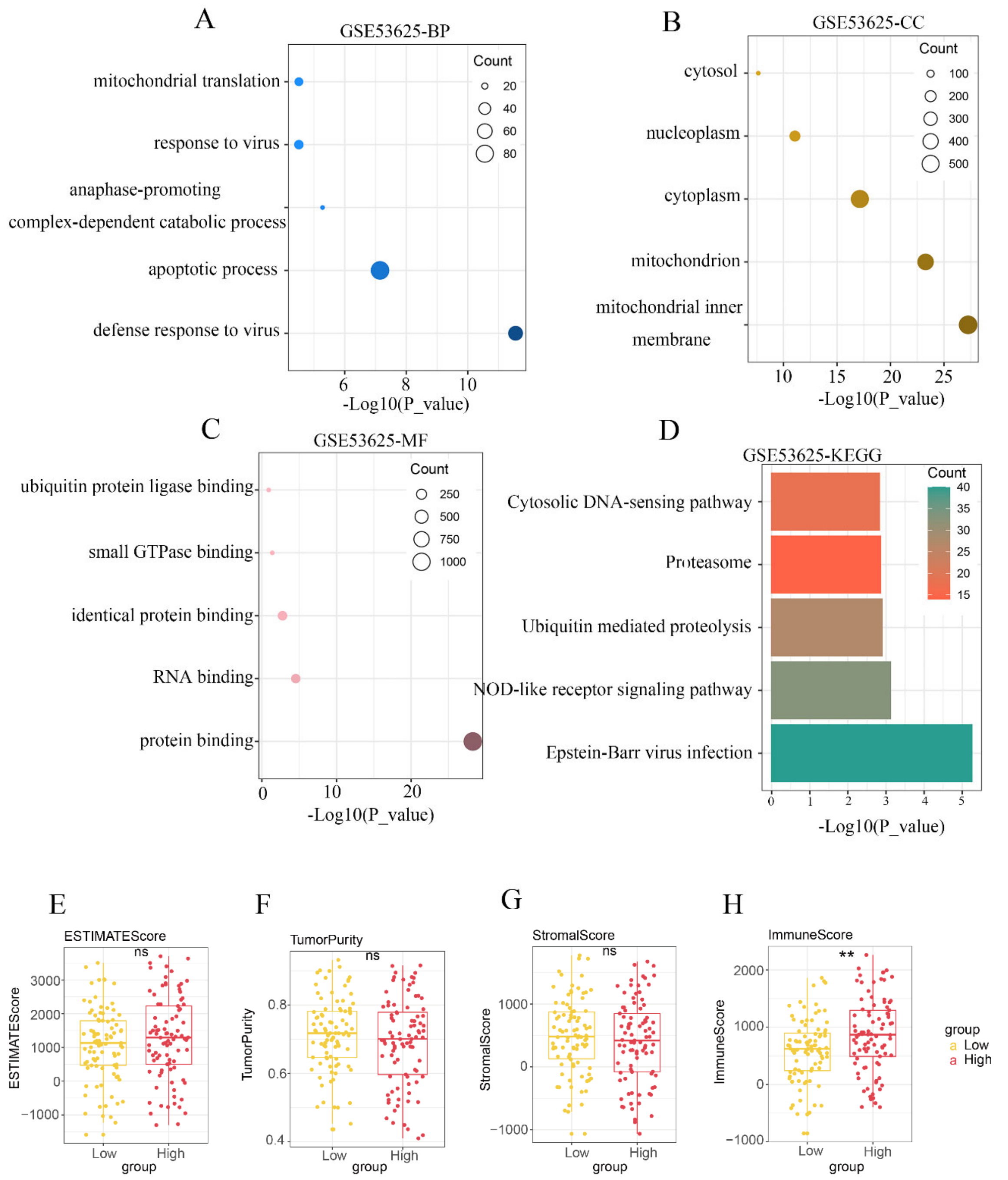

Figure S2: Immunological and functional enrichment analysis of the GSE53625 cohort. GO enrichment analysis of Biological Process (BP)(A), Cellular Component (CC)(B), and Molecular Function (MF)(C) categories in the GSE53625 cohort, with significant terms displayed based on their

-log10(adjust-p). Circle size represents the number of enriched genes, and color intensity reflects the significance of the enrichment. BP terms, including mitochondrial translation, response to virus, and apoptotic process(A). CC terms, including cytosol, nucleoplasm, and mitochondrial inner membrane(B). MF terms, including protein binding, RNA binding, and ubiquitin-protein ligase binding(C). KEGG pathway analysis highlights the most enriched signaling pathways in the GSE53625 cohort, such as the Cytosolic DNA-sensing pathway and Epstein-Barr virus infection pathway(D). The x-axis indicates −log10(FDR) values based on Benjamini–Hochberg adjusted P values, and the y-axis lists the enriched terms. Boxplots showing StromalScore (E), TumorPurity (F), StromalScore (G), and ImmuneScore (H) between high and low *NLRC5* expression groups. Significant differences are indicated, and high expression groups demonstrate distinct immune profiles.

A

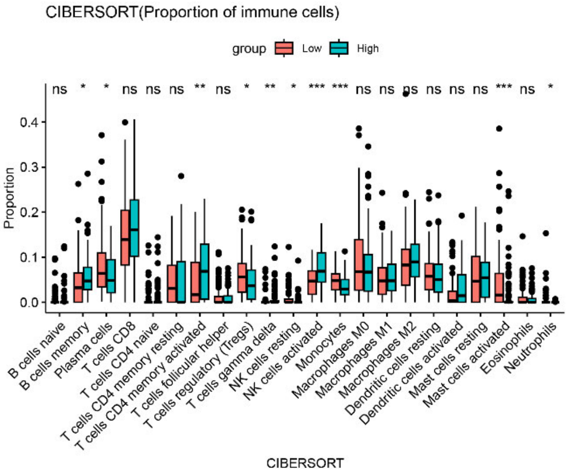

B

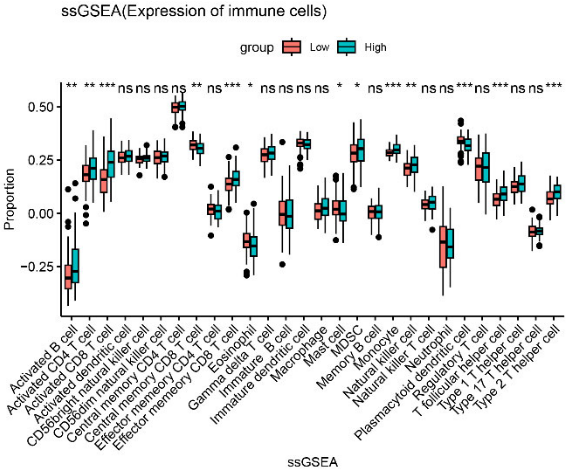

Figure S3: **Comparison of immune cell proportions between *NLRC5*-high and *NLRC5*-low expression groups in the GSE53625 cohort.** CIBERSORT analysis showing the proportion of various immune cell types between *NLRC5*-high (cyan) and *NLRC5*-low (red) groups(A). Significant differences in immune cell infiltration are observed for multiple cell types, including increased levels of activated $CD8^{+}$T cells, M1 macrophages, and T follicular helper cells in the *NLRC5*-high group. ssGSEA (single-sample Gene Set Enrichment Analysis) results showing the expression of immune-related gene sets(B). *NLRC5*-high tumors display a significant enrichment of immune cell populations such as activated $CD8^{+}$T cells, activated dendritic cells, and M1 macrophages, reinforcing the role of *NLRC5* in modulating the immune microenvironment of ESCC. Significance levels are indicated as *$p < 0.05$, **$p < 0.01$, ***$p < 0.001$, and ns for no-significant. (n= 179)

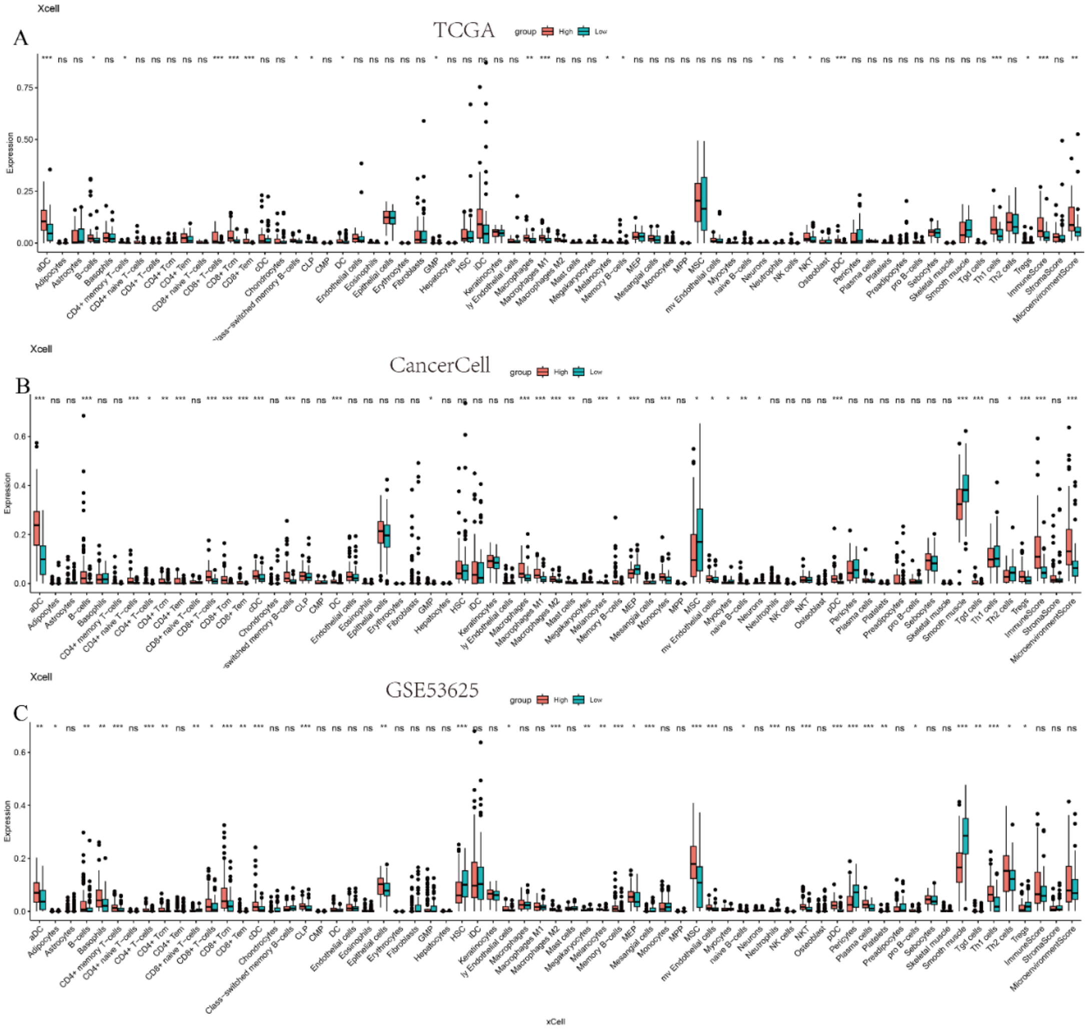

Figure S4: Using the Xcell immune analysis method, the differences in immune cell abundance between high and low *NLRC5* expression groups were analyzed in three cohorts: TCGA, Cancer Cell, and GSE53625. (A) represents the TCGA cohort(n = 80), (B) represents the Cancer Cell cohort(n = 155), and (C) represents the GSE53625 cohort(n = 179). Significance levels are indicated as *p < 0.05, **p < 0.01, ***p < 0.001, and ns for no-significant.

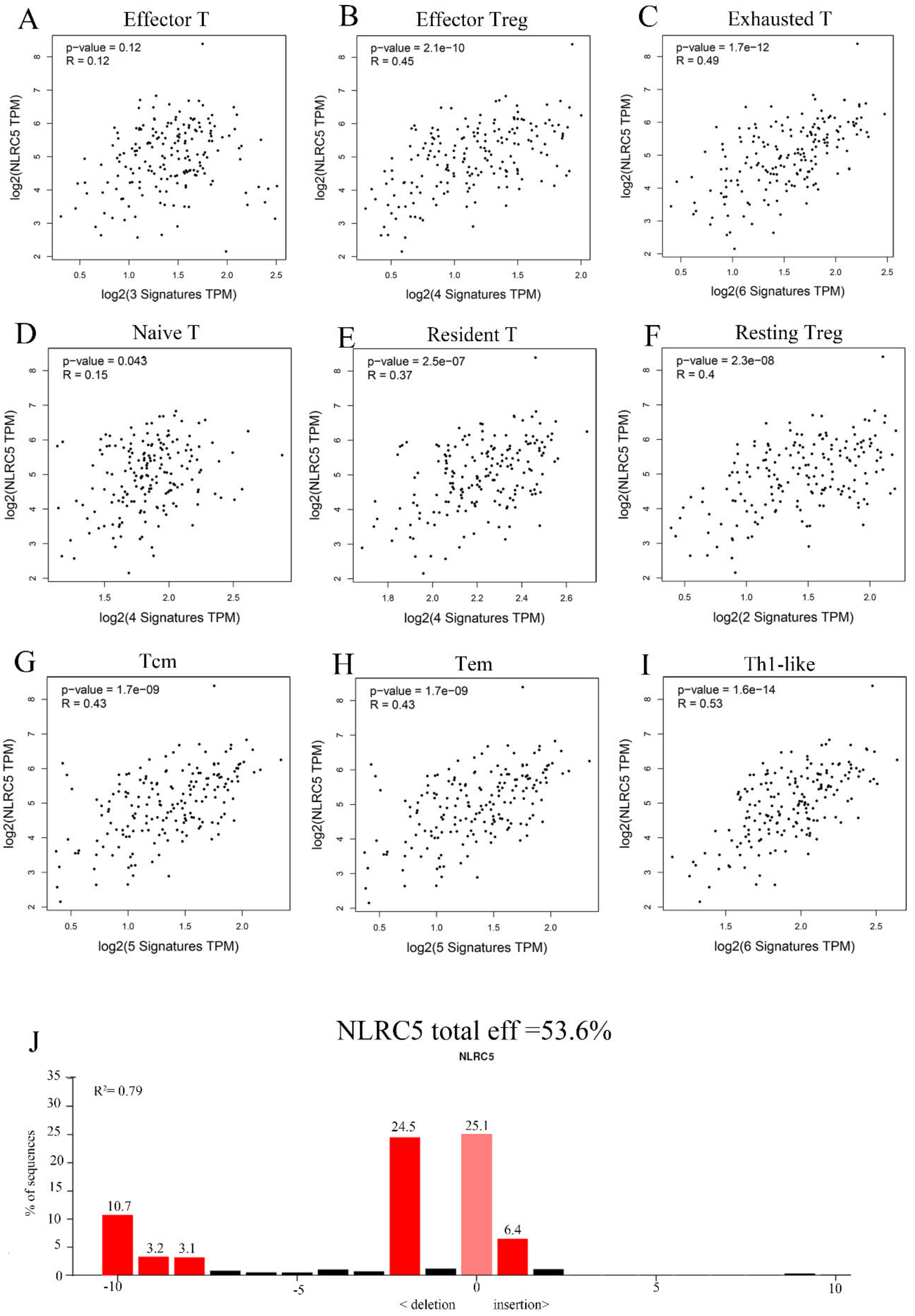
A
Effector T
p-value = 0.12
R = 0.12
log2(NLRC5 TPM)
log2(3 Signatures TPM)
B
Effector Treg
p-value = 2.1e−10
R = 0.45
log2(NLRC5 TPM)
log2(4 Signatures TPM)
C
Exhausted T
p-value = 1.7e−12
R = 0.49
log2(NLRC5 TPM)
log2(6 Signatures TPM)
D
Naive T
p-value = 0.043
R = 0.15
log2(NLRC5 TPM)
log2(4 Signatures TPM)
E
Resident T
p-value = 2.5e−07
R = 0.37
log2(NLRC5 TPM)
log2(4 Signatures TPM)
F
Resting Treg
p-value = 2.3e−08
R = 0.4
log2(NLRC5 TPM)
log2(2 Signatures TPM)
G
Tcm
p-value = 1.7e−09
R = 0.43
log2(NLRC5 TPM)
log2(5 Signatures TPM)
H
Tem
p-value = 1.7e−09
R = 0.43
log2(NLRC5 TPM)
log2(5 Signatures TPM)
I
Th1-like
p-value = 1.6e−14
R = 0.53
log2(NLRC5 TPM)
log2(6 Signatures TPM)
J
NLRC5 total eff =53.6%
NLRC5
R²= 0.79
% of sequences
10.7
3.2
3.1
24.5
25.1
6.4
< deletion
insertion>

Figure S5: Correlation between *NLRC5* expression and T cell–related transcriptional signatures. (A) Effector T cell signatures showed a weak and non-significant correlation with *NLRC5* expression. (B) Effector regulatory T (Treg) cells exhibited a moderate positive correlation with *NLRC5* expression. (C) Exhausted T cell signatures demonstrated a strong positive correlation with *NLRC5* expression. (D) Naive T cell signatures displayed a weak but statistically significant positive correlation with *NLRC5* expression. (E) Resident T cell signatures were positively correlated with *NLRC5* expression. (F) Resting Treg signatures showed a significant moderate positive correlation with *NLRC5* expression. (G) Central memory T (Tcm) cell signatures were moderately correlated with *NLRC5* expression. (H) Effector memory T (Tem) cell signatures exhibited a moderate positive correlation with NLRC5 expression. (I) Th1-like cell signatures showed a strong positive correlation with *NLRC5* expression. (J): TIDE analysis of *NLRC5* gene editing efficiency in primary human T cells. The x-axis indicates the size of insertions or deletions relative to the wild-type sequence, while the y-axis represents the percentage of total sequencing reads. Indel frequencies at the *NLRC5* locus following CRISPR/Cas9 editing were quantified by TIDE analysis.

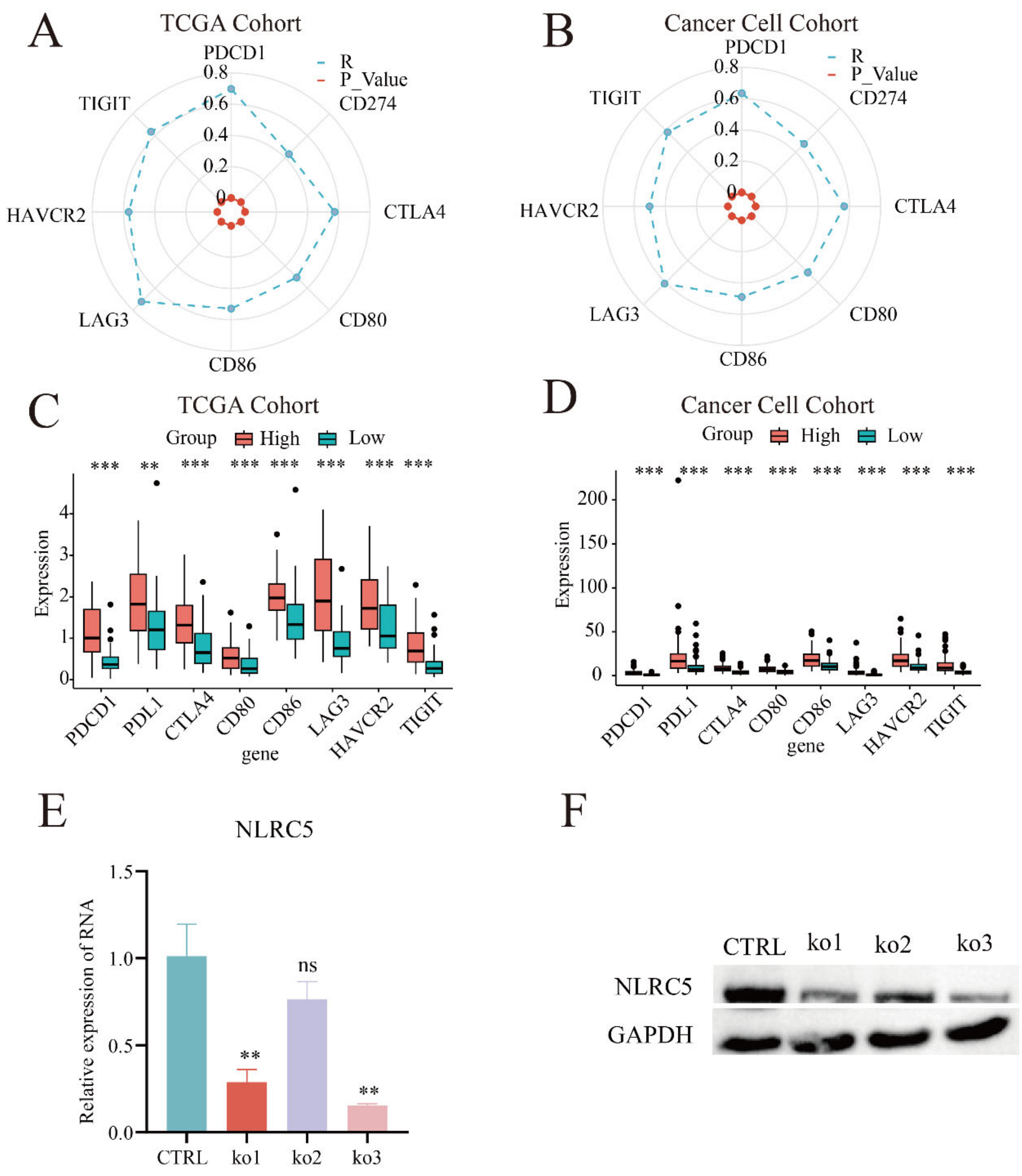

Figure S6 Association between *NLRC5* and immune checkpoint molecules and validation of NLRC5 knockout in T cells.(A–B) Radar plots showing the correlation between *NLRC5* expression and immune checkpoint–related genes (*PDCD1, CD274, CTLA4, CD80, CD86, LAG3, HAVCR2*, and *TIGIT*) in the TCGA cohort (A) and the CancerCell cohort (B). Blue dashed lines indicate Pearson correlation coefficients (R), and red points indicate the corresponding p-values.(C–D) Expression levels of immune checkpoint–related genes in the *NLRC5* high and low expression groups in the TCGA cohort (C) and CancerCell cohort (D). Gene expression differences between groups were compared using the Wilcoxon rank-sum test. Data are shown as boxplots indicating median and interquartile range.(E) Quantitative PCR analysis showing relative *NLRC5* mRNA expression in control (CTRL) and CRISPR/Cas9-mediated *NLRC5* knockout (KO) primary human T cells using three independent sgRNAs (ko1–ko3).(F) Western blot analysis confirming *NLRC5*

protein depletion in NLRC5-KO T cells compared with CTRL cells. GAPDH was used as a loading control. Statistical significance is indicated as follows: ns, not significant; ** $p < 0.01$; *** $p < 0.001$.
